# Age-related deregulation of TDP-43 after stroke enhances NF-κB-mediated inflammation and neuronal damage

**DOI:** 10.1186/s12974-018-1350-y

**Published:** 2018-11-09

**Authors:** Sai Sampath Thammisetty, Jordi Pedragosa, Yuan Cheng Weng, Frédéric Calon, Anna Planas, Jasna Kriz

**Affiliations:** 1 CERVO Brain Research Centre, Université Laval, 2601 Chemin de la Canardière, Québec, QC G1J 2G3 Canada; 2 IDIBAPS, Barcelona, Spain; 3 Research Centre of the CHUQ, Université Laval, Québec, QC G1J2G3 Canada; 4 Faculty of Pharmacy, Université Laval, Québec, QC G1J2G3 Canada; 5 Department of Psychiatry and Neuroscience, Faculty of Medicine, Université Laval, 2601 Chemin de la Canardière, Québec, QC G1J2G3 Canada

**Keywords:** Post-stroke inflammation, Aging, Acute neurodegeneration, Microglia, Innate immune response, Neuronal injury

## Abstract

**Background:**

TDP-43 has been identified as a disease-associated protein in several chronic neurodegenerative disorders and increasing evidence suggests its potentially pathogenic role following brain injuries. Normally expressed in nucleus, under pathological conditions TDP-43 forms cytoplasmic ubiquitinated inclusions in which it is abnormally phosphorylated and cleaved to generate a 35 and a 25 kDa C-terminal fragments. In the present study, we investigated age-related expression patterns of TDP-43 in neurons and glia and its role as modulator of inflammation following ischemic injury.

**Methods:**

Wild-type and TDP-43 transgenic mice of different age groups were subjected to transient middle cerebral artery occlusion. The role of TDP-43 in modulation of inflammation was assessed using immunofluorescence, Western blot analysis, and in vivo bioluminescence imaging. Finally, post-mortem stroke human brain sections were analyzed for TDP-43 protein by immunohistochemistry.

**Results:**

We report here an age-related increase and formation of ubiquitinated TDP-43 cytoplasmic inclusions after stroke. The observed deregulation in TDP-43 expression patterns was associated with an increase in microglial activation and innate immune signaling as revealed by in vivo bioluminescence imaging and immunofluorescence analysis. The presence of ubiquitinated TDP-43 aggregates and its cleaved TDP-35 and TDP-25 fragments was markedly increased in older, 12-month-old mice leading to larger infarctions and a significant increase in in neuronal death. Importantly, unlike the hallmark neuropathological features associated with chronic neurodegenerative disorders, the TDP-43-positive cytoplasmic inclusions detected after stroke were not phosphorylated. Next, we showed that an increase and/or overexpression of the cytoplasmic TDP-43 drives the pathogenic NF-κB response and further increases levels of pro-inflammatory markers and ischemic injury after stroke in age-dependent manner. Finally, analyses of the post-mortem stroke brain tissues revealed the presence of the cytoplasmic TDP-43 immunoreactive structures after human stroke.

**Conclusion:**

Together, our findings suggest that the level of cytoplasmic TDP-43 increases with aging and may act as an age-related mediator of inflammation and neuronal injury after stroke. Thus, targeting cytoplasmic TDP-43 may have a therapeutic potential after stroke.

**Electronic supplementary material:**

The online version of this article (10.1186/s12974-018-1350-y) contains supplementary material, which is available to authorized users.

## Background

Stroke is a leading cause of death and the major cause of long-lasting disabilities in industrialized countries ([1]). Indeed, patients surviving stroke will carry a major risk for development of vascular and/or Alzheimer’s style dementia later in the life [2–4]. This risk is particularly elevated in elderly population as various cellular processes are altered in aging. Aging hampers the normal physiology of the cell, leading to a metabolic dysfunction, oxidative stress, inflammation, and/or DNA damage [5–7]. Growing evidence suggests that aging in the brain is associated with a progressive loss of immune homeostasis (a chronic low-level inflammation) leading to an overall increase in the pro-inflammatory cytokines including IL-1β, TNF-α [7, 8]. In keeping with previous evidence, we recently demonstrated that processes associated with aging significantly affect microglia activation patterns and innate immune signaling after stroke in both female and male mice [9, 10]. In particular, we observed a marked deregulation of Toll-like receptor 2 (TLR2) induction patterns in activated microglia followed by alterations in the innate immune downstream signaling events and larger infarctions [9, 10]. However, how aging affects immune signaling in neurons and/or microglia/neurons crosstalk in response to ischemic injury remains unclear.

In a search for proteins that may affect microglia/neuron immune crosstalk in aging brain, we focused our study on transactive response (TAR) DNA binding protein 43 (TDP-43). Generally localized in the nucleus, TDP-43 belongs to the family of heterogeneous nuclear ribonuclear proteins that are highly conserved in different species [11]. TDP-43 regulates gene expression by controlling several processes such as pre-mRNA splicing [11], mRNA stabilization [12], mRNA transport, and translation [13]. TDP-43 has been identified as a major constituent of ubiquitinated nuclear and cytoplasmic inclusions in frontotemporal lobar degeneration [14], ALS [15] and Alzheimer’s disease [16, 17]. Normally, localized in the nucleus, under pathological conditions TDP-43 forms insoluble ubiquitinated inclusions in which it is abnormally phosphorylated and cleaved to generate a 35 and a 25 kDa C-terminal fragments lacking the N-terminus nuclear localization signal [15, 18]. In addition to processes associated with chronic neurodegeneration, increasing evidence suggests that deregulation of TDP-43 neurons may occur following brain injuries including single and repetitive traumatic brain injury (TBI) [19, 20], while Uchino and colleagues recently reported presence of TDP-43-positive inclusions in aging brains [21]. To date, the molecular mechanisms by which TDP-43 may induce neurodegeneration and neuronal death remain elusive. However, our previous work suggests that TDP-43 may serve as a modulator of inflammation, acting as co-activator of p65 NF-κB [22]. Here, we hypothesized that gradual age-related accumulation of cytoplasmic TDP-43 may trigger activation of NF-κB pathogenic pathways, leading to a deregulation of innate immune response and thus increasing susceptibility of neurons to ischemic injury.

The current study was designed to identify and characterize the age-related expression patterns of TDP-43 in neurons and microglia and to evaluate its role as modulator of inflammation following ischemic injury. We report here an age-related increase and long-lasting mislocalization of TDP-43 after stroke. The observed accumulation of cytoplasmic TDP-43 was associated with an increase in microglial activation and innate immune signaling seen by in vivo bioluminescence imaging and immunofluorescence analysis. The presence of ubiquitinated TDP-43 aggregates and its cleaved TDP-35 and TDP-25 fragments was markedly increased in older, 12-month-old mice, which showed larger infarctions alongside with an increase in neuronal death. We next showed that increase and/or overexpression of the cytoplasmic TDP-43 drives the NF-κB response and further increase levels of pro-inflammatory markers and ischemic injury after stroke. Overall, our results suggest that TDP-43 may act as an age-related modulator of inflammation after stroke. Based on our results, we propose that therapies targeting cytoplasmic TDP-43 may have a potential to modulate post-ischemic inflammation and to protect dying neurons in the ischemic microenvironment. Of note, the post-mortem analysis of the brains autopsied at different time points after human stroke suggests the presence of TDP-43 immunoreactive structures localized in the cytoplasm of the neurons in periphery and the core region of the ischemic lesion.

## Materials and methods

### Animals

The wild-type (C57Bl/6) mice of 3 and 12 month old (representing a middle aged mouse group) were selected for study. The TLR2-luc-GFP transgenic reporter mice were developed, validated, and genotyped as described previously [23]. These animals do not develop any overt phenotype and were used for in vivo bioluminescence imaging analysis of microglia activation/innate immune response. The TDP-43 A315T transgenic mice were generated, described, and genotyped as described in [24]. The TDP-43 A315T mice develop age-related cognitive deficits resembling frontotemporal dementia phenotypes. All our transgenic colonies are kept in C57Bl/6 genetic background. All experimental animals used in this study were provided with water and healthy diet and were monitored during the entire experimental protocol. The animals were held in the pathogen-free animal facility of the CERVO Brain Research Institute, 3–5 mice per cage in the controlled environment having the 12 h day and night cycles. To avoid the biological effects of sex on ischemic injury, the experiments were performed on male mice. All the experimental procedures were approved by the Laval University Animal care Ethics Committee and are in accordance with the *Guide to the Care and Use of Experimental Animals* of the Canadian Council on Animal Care.

### Surgical procedure

Transient focal cerebral ischemia was induced by unilateral left middle cerebral artery occlusion (MCAO) as described [23]. Wild-type mice of around 3–12 months were selected and unilateral transient focal cerebral ischemia was induced by intraluminal filament occlusion of the left middle cerebral artery (MCA) with a 6–0 silicone-coated monofilament suture for 1 h followed by reperfusion times of 24 h, 48 h, 72 h, 5 days, and 10 days after surgery. The body temperature was maintained at 37 °C with a heating pad.

### In vivo bioluminescence imaging

The images were obtained by using IVIS 200 imaging system (Caliper LS-Xenogen, Alameda, CA, USA). Twenty minutes prior to imaging session, the mice were administered with d-luciferin (150 mg/kg bw), a substrate for luciferase dissolved in 0.9% saline. The mice were then anesthetized in 2% isoflurane in 100% oxygen at a flow rate of 2 L/min, placed in a heated light-tight imaging chamber. All the animals were imaged before for baseline expression and then continued at different time points post MCAO. Images were captured using a high sensitivity CCD camera with wavelengths ranging from 300 to 600 nm and exposure time for imaging of brain was set for 1 min. The bioluminescence emission was quantified by determining the total number of photons emitted per second (p/s) using live image 2.5 acquisitions and imaging software. Region of interest measurements were used to convert surface radiance (p/s/cm^2^/sr) to source flux or the total flux of photons expressed in photons/second. The data are represented as pseudo-color images indicating light intensity (red and yellow, most intense), which were superimposed over gray-scale reference photographs [25].

### Protein lysate preparation and immunoblots

Cytoplasmic and nuclear fractions were obtained as per the protocol described earlier [26, 27]. Five hundred milligrams of fresh brain tissue samples were transferred to 1 ml of cell lysis buffer (10 mM HEPES, 10 mM NaCl, 1 mM KH_2_PO_4_, 5 mM MgCl_2,_ phosphatase, and protease inhibitors), and homogenized by applying two strokes in a glass homogenizer. The suspension was incubated for 10 min on ice and then homogenized by applying six strokes in a motorized homogenizer at 250 rpm. Differential centrifugation was performed after restoration with 100 μl of 2.5 M sucrose. The first round of centrifugation was performed at 6300×*g* for 10 min at 4 °C. The pellet was collected and suspended in TSE buffer (10 mM Tris, 300 mM sucrose, 1 mM EDTA, 0.1% Non idet P-40, 10× *v*/*w*, phosphatase, and protease inhibitors pH 7.5) and homogenized with 30 strokes using a motorized Teflon potter at 250 rpm. The obtained suspension was centrifuged at 4000×*g* for 5 min. The resulting supernatant was discarded, and the pellet was washed with TSE buffer until the supernatant was clear. Pellet was then suspended in RIPA buffer (50 mM Tris, 1 mM EDTA, 150 mM Nacl, 0.1% SDS, 1% Non-idet P-40, 0.5% sodium deoxycholate, phosphatase, and protease inhibitors) with 2% SDS as nuclear fraction. The supernatant collected from the first round of differential centrifugation was subjected for centrifugation at 10,700 g for around 30 min and the supernatant collected was used as cytoplasmic fraction. The protein lysates from different fractions were quantified by Bradford and subjected for Western blot and co-immunoprecipitation as described previously [24].

### Tissue collection and immunohistochemistry

The mice were anesthetized and perfused transcardially with phosphate buffer solution followed by 4% paraformaldehyde at pH 7.4. The brain tissue was post fixed overnight in 4% paraformaldehyde and then cryo-preserved in 30% sucrose. The following procedure was adopted for the study as described earlier [24]. The fixed brains were sliced into 25 μm sections, washed with phosphate buffer saline thrice, and blocked with 5% goat serum for 1 h. The sections were incubated over night with respective primary antibodies—rabbit polyclonal TDP-43 (Protein tech, IL, USA, 1:1000), mouse monoclonal NeuN (Millipore; 1:1000), rabbit polyclonal Iba1 (WAKO; 1:500), rat monoclonal CD11b (serotech 1:500), and caspase-3 (Cell signaling; 1:100), mouse monoclonal ubiquitin (Millipore; 1:500) followed by incubation with respective fluorescent goat Alexa Fluor 488 and 594 (1:500) secondary antibodies (Invitrogen) for 2 h at room temperature. Finally, the microscopic images were captured using confocal microscope (Zeiss) and Apotome (Zeiss Axio vision).

### Infarct size

The mice were anesthetized and perfused transcardially using phosphate buffer saline followed by 4% paraformaldehyde solution (pH 7.4). The brains were then sectioned to 25-μm-thick slices and stained with cresyl violet histological stain. The mean stroke area of approximately ten sections from 3 to 12 months old were calculated after 72 h post MCAO by using ImageJ software and expressed as % stroke area. (%Stroke area = (infarct size / total contra lateral side of the section) × 100) [28].

### Cytokine array

The mouse cytokine array (Ray bio Mouse cytokine Antibody Array, C1 series, Ray bio, Inc.) was used to detect the levels of different cytokines in sham, acute, chronic stroke-operated mice. The array was performed according to the manufacturer’s instructions. The protein lysates were obtained by homogenization of brains of from respective groups using 1x cell lysis buffer (provided in the kit). The protein concentration was determined for each sample and diluted to 300 μg in 1x blocking buffer. Samples for each group (three mice/group) were pooled and incubated with array membrane overnight at 4 °C. After washes, the membranes were incubated with biotin conjugated primary antibody provided in the kit overnight at 4 °C and next day following successive washes, membranes were incubated with secondary antibody provided for 2 h at room temperature. As per the ray biotech protocol, protein levels were visualized by chemiluminescence and quantified using ImageJ software. The protein levels on each array were standardized against an internal positive control on the array (Lalancette et al. 2007; Bohacek et al. 2012).

### Immunohistochemistry/human tissue

As described previously [29], paraffin-embedded human brain sections of 5 μm from the frontal cortical lobe were examined to evaluate reactivity towards TDP-43 protein. The paraffin-embedded sections were deparaffinized in xylene and rehydrated in a descending series of ethanol. Endogenous peroxidases were blocked with 5% hydrogen peroxide in methanol, and antigen retrieval was carried out using sodium citrate buffer for 30 min. Sections were then blocked by using goat serum and incubated overnight with anti-TDP-43 (abnova: E2 clone 1:1000). Next, labeling was detected by using species-specific biotinylated the EnVision™ + System, Peroxidase (Dako, Agilent). Slides were cover slipped using DPX mounting medium (#06522, Sigma). For the used tissues, we obtained written consent from the families for tissue removal after death for diagnostic and research purposes at the Neurological Tissue bank of the Biobank-Hospital Clínic-Institut d’Investigacions Biomèdiques August Pi i Sunyer (IDIBAPS). We also obtained tissue from control subjects stored at this Biobank. The study had the approval of the Ethics Committee of Hospital Clínic de Barcelona (CEIm). Features of the cases are shown in Additional file 1: Table S1.

### Statistical analysis

The data quantified and represented as mean ± SEM. Statistical analysis was carried using Prism 7 (Graph Pad Software, La Jolla, CA, USA). Comparison between two groups was achieved by unpaired *t* test and comparisons between multiple groups were done using one-way ANOVA followed by Tukey’s post-hoc multiple comparison test. Statistical significance was defined when ***p < 0.001, **p < 0.01, *p < 0.05. In all experimental procedures, *n* (per group) = 5–10 animal.

## Results

### Increase in cytoplasmic TDP-43 and pathological TDP-35, TDP-25 fragments in the 12-month-old mice after stroke

**Fig. 1:**
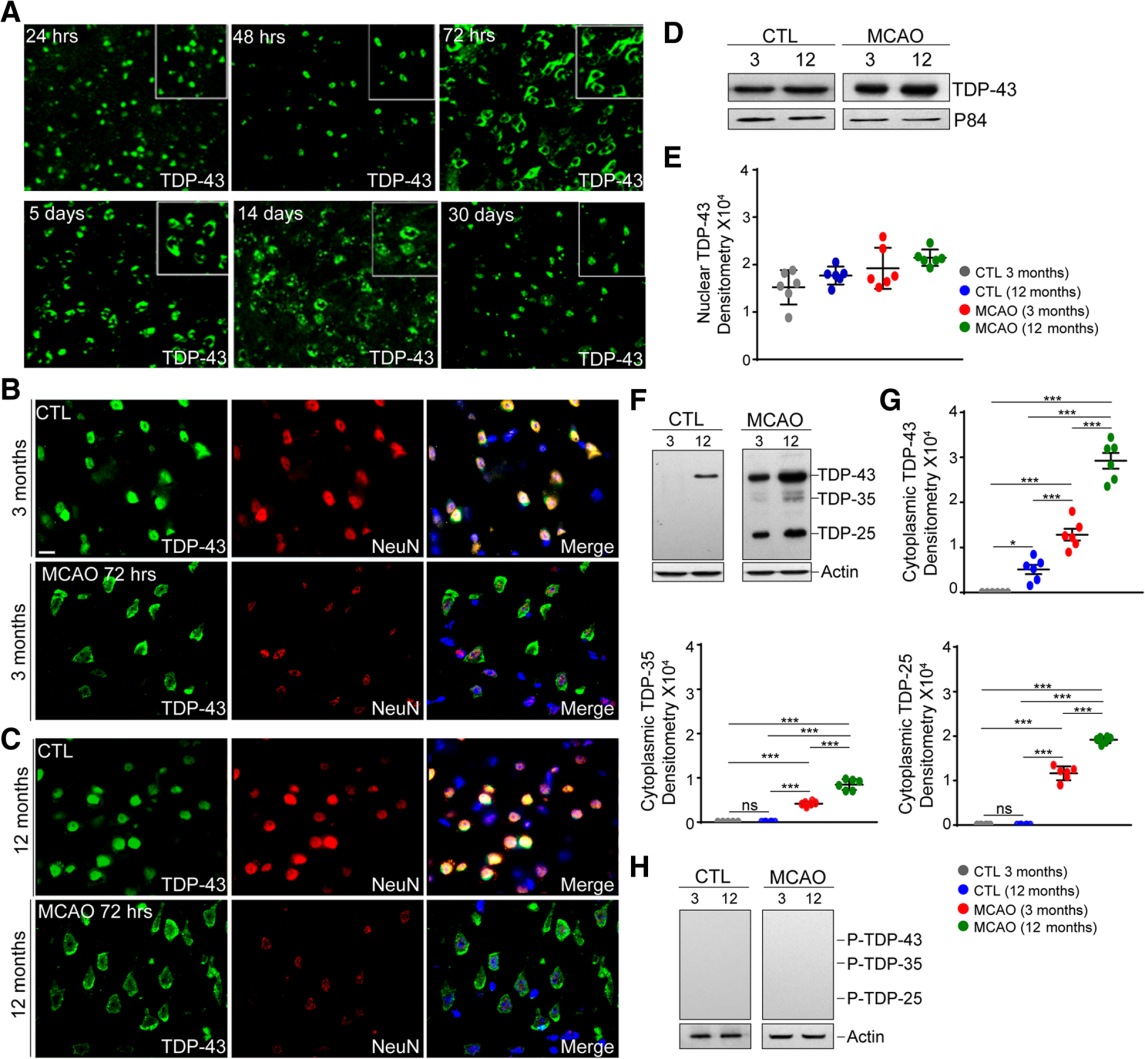
Characterization of TDP-43 expression patterns in aging and stroke. **a** Immunofluorescence of the brain cortex of wild-type 3-month-old mice using TDP-43 antibody at different time points after MCAO reveals mislocalization of TDP-43 protein into cytoplasm starting 72 h after MCAO. **b** Double immunofluorescence of the brain cortex sections of 3- and **c** 12-month-old mice in control conditions CTL (upper panels) and 72 h after MCAO using TDP-43 antibody (green) and NeuN antibody (red) show nuclear localization of TDP-43 in control conditions and cytoplasmic mislocalization of TDP-43 in neuronal cells in both age groups. **d**, **e** Western blot of nuclear lysates from 3- and 12-month-old mice using TDP-43 antibody in control and 72 h post MCAO does not reveal any significant changes in the levels of whole length TDP-43. P84 is used as loading control. **f** Western blot of cytoplasmic lysates from 3- and 12-month-old mice using TDP-43 antibody in control and 72 h after MCAO show expression of whole length TDP-43, fragmented TDP-35, and TDP-25. **g** Western blot of cytoplasmic lysates from 3- and 12-month-old mice using phospho-TDP-43 antibody in control and 72 h after MCAO show no expression of whole length P-TDP-43, fragmented P-TDP-35, or P-TDP-25. **h** Normalized densitometry values of immunoblots from control and 72 h after MCAO reveal significant increase in the levels of whole length TDP-43, pathological TDP-35, and TDP-25 fragments in aging. Actin is used as loading control. Quantified data in the figure was presented as mean ± SEM and statistical significance between the groups was achieved using ANOVA followed by Tukey’s multiple comparison test and depicted as ***p < 0.001. Scale bar represents 10 and 20 μm

The presence of pathological TDP-43-positive inclusions in cytoplasm and nucleus of both the neurons and glia in ALS, FTLD, and Alzheimer’s has been widely established [17, 24]. Moreover, growing evidence suggests that TDP-43 may have a role in the acute neurodegeneration/neuroinflammation triggered by different types of brain injuries including TBI and stroke. To better understand the role of TDP-43 in the brain response to ischemic injury, we first characterized the expression pattern of TDP-43 by immunofluorescence staining employing anti-TDP-43 antibody in the wild-type (WT) mice that were subjected to transient MCAO followed by different reperfusion periods. While within the first 24 h after stroke TDP-43 expression was mostly limited to the nuclei, at 48 h after MCAO, few cells start to show cytoplasmic TDP-43, while at 72 h post-stroke till the latest time point analyzed (30 days), most of the cells displayed strong cytoplasmic TDP-43 immunoreactivity (Fig. 1a). Because incidence of stroke increases with aging (peaking at middle age time), we next performed MCAO on 3- and 12-month-old WT mice and asked whether TDP-43 subcellular distribution and expression patterns in control conditions and after stroke are affected by processes of aging. Normally, TDP-43 is a nuclear protein (Fig. 1b, c upper panels) and as shown in Fig. 1d, e, the expression of whole-length TDP-43 from the nuclear lysates isolated from control brains and 72 h post MCAO mice did not show significant difference between 3 and 12 month mice. Because brain injury may trigger formation of the pathological TDP-43 species including TDP-35 and TDP-25 C-terminal fragments, we next asked whether aging may contribute to a formation of pathological TDP-43 species. To address this issue, we analyzed the expression patterns of TDP-43, TDP-35, and TDP-25 fragments in the cytoplasmic lysate collected 72 h post MCAO. While ischemic injury caused mislocalization of TDP-43 into cytoplasm in both age groups (Fig. 1b, c, f), Western blot analysis revealed a significant increase in the expression of whole-length cytoplasmic TDP-43 in the ischemic brains of 12 months compared to 3-month-old mice and corresponding controls (Fig. 1f, g). The same expression pattern was observed for pathological TDP-35 and TDP-25 fragments. As further revealed in Fig. 1f, ischemic injury in older, 12-month-old animals was associated with significantly higher increase in the expression of pathological cytoplasmic TDP-35 and TDP-25 fragments when compared to 3 month old (Fig. 1f, g). Importantly, the pathological TDP-35 and TDP-25 fragments were not detected in the cytoplasmic lysates from the brains of 3- and 12-month-old controls, non-stroked mice (Fig. 1f, g). Of note, a low amount of full-length cytoplasmic TDP-43 was detected in the brains of non-stroked 12-month-old mice. Taken together, our results suggest that cytoplasmic mislocalization of TDP-43 after stroke may represent a common mechanism of the neuronal response to ischemic injury while formation and expression of pathogenic, truncated TDP-35 and TDP-25 species suggests an age-related process. Importantly, as shown in Fig. 1h, unlike the chronic neurodegeneration, acute brain ischemia did not trigger formation of phosphorylated TDP-43 aggregates.

### Formation of TDP-43-ubiquitin aggregates in neurons and TDP-43 mislocalization in microglial cells after MCAO is enhanced in older mice

**Fig. 2:**
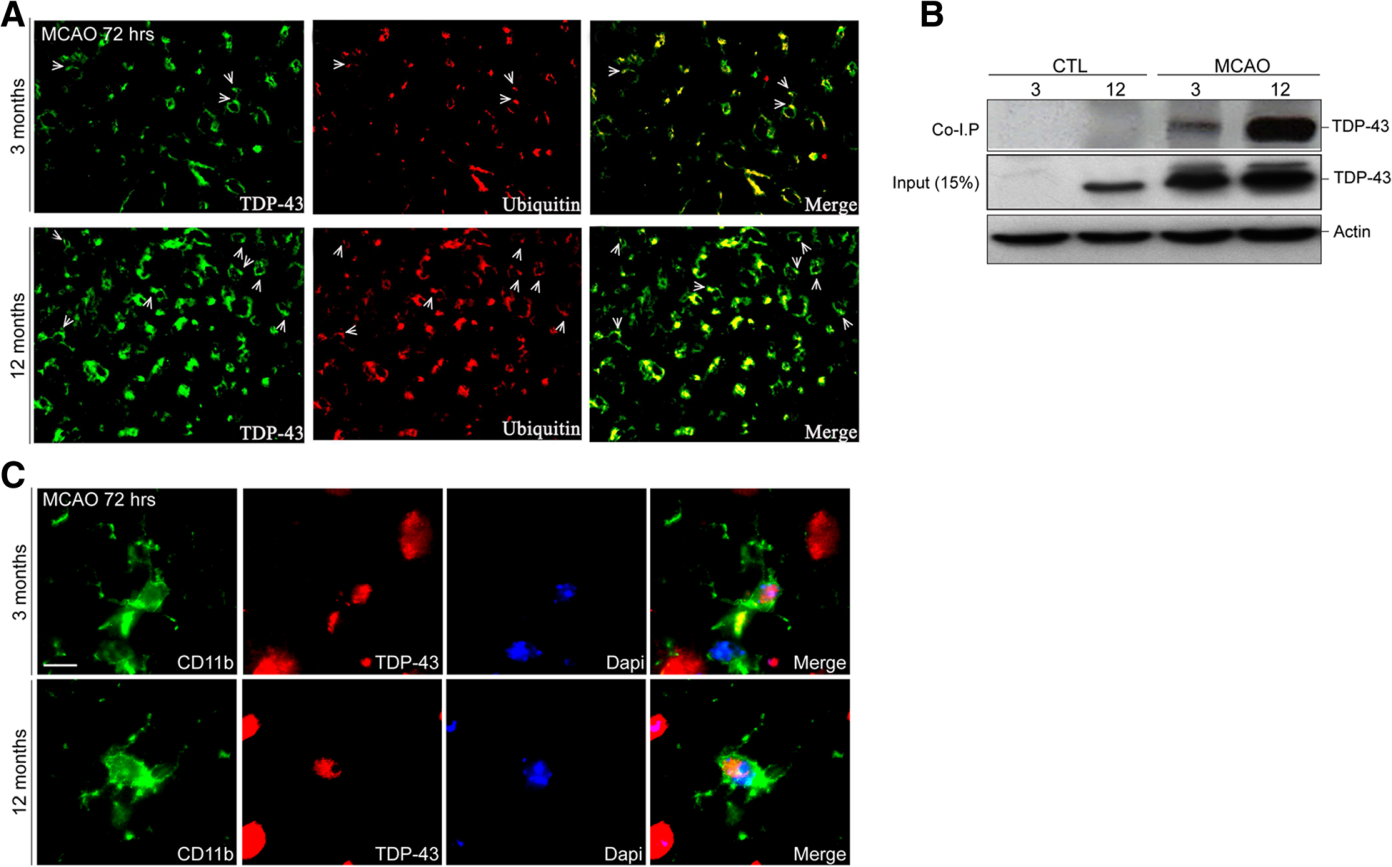
Cytoplasmic TDP-43 shows ubiquitin positivity and microglia/macrophages exhibit TDP-43 mislocalization. **a** Double immunofluorescence of the brain cortex sections of 3- and 12-month-old mice 72 h after MCAO using TDP-43 antibody (green) and ubiquitin (red) reveal the formation of cytoplasmic TDP-43-ubiquinated aggregates 72 h after MCAO in 3- and 12-month-old mice. **b** Co-immunoprecipitation of ubiquitin using rabbit polyclonal TDP-43 from the cytoplasmic lysates of 3- and 12-month-old control and MCAO operated mice reveal the ubiquitination of TDP-43 after 72 h post MCAO. Note that TDP-43 ubiquitination is increased in 12-month-old transgenic mice after MCAO. Western blot of TDP-43 using rabbit polyclonal TDP-43 is shown as 15% input to confirm the presence of TDP-43 protein in the cytoplasmic lysate we used for Co-immunoprecipitation and actin is shown as loading control. **c** Double immunofluorescence of the brain cortex sections of 3- and 12-month-old mice 72 h after MCAO using TDP-43 antibody (red) and CD11b antibody (green) show mislocalization of TDP-43 in microglial cells in both age groups. Scale bar represents 10 μm

The main histological feature of neurodegenerative disorders such as ALS/FTLD and to a lesser extent AD is a presence of ubiquitinated TDP-43 immunoreactive cytoplasmic inclusions in neuronal and glial cells. In order to determine if cerebral ischemia increases the level of ubiquitination of TDP-43 in an age-dependent manner, we performed a double immunofluorescence analysis using anti-TDP-43 and anti-ubiquitin antibodies on 3- and 12-month-old control mice and 72 h after MCAO. As shown in Fig. 2a, a double immunofluorescence analysis revealed a colocalization of ubiquitin staining with the cytoplasmic TDP-43 forming a ring-shaped TDP-43/ubiquitin aggregates. The TDP-43/ubiquitin aggregates were present in the mice of both age groups (Fig. 2a); however, the number of TDP-43/ubiquitin positive inclusions seems to be higher in the ischemic brains of the 12 months mice. These results obtained by immunofluorescence were further confirmed by co-immunoprecipitation experiments of TDP-43 protein with ubiquitin (Fig. 2b). The immunoprecipitation experiments clearly demonstrated that TDP-43 is highly ubiquitinated 72 h after MCAO. The intensity of ubiquitination of TDP-43 was more pronounced in the brains of 12-month-old mice compared to the 3-month-old mice 72 h after MCAO. Importantly, the control samples of both the age groups were devoid of ubiquitinated TDP-43 protein. Next, we asked whether ischemic injury triggers cytoplasmic mislocalization of TDP-43 in glial cells. As further revealed in Fig. 2c, we observed few microglial cells showing cytoplasmic TDP-43 72 h post MCAO in both age groups suggesting that deregulation of TDP-43 is not only confine to neurons after acute stroke.

### Enhanced inflammatory response in aging mice following ischemic injury

**Fig. 3:**
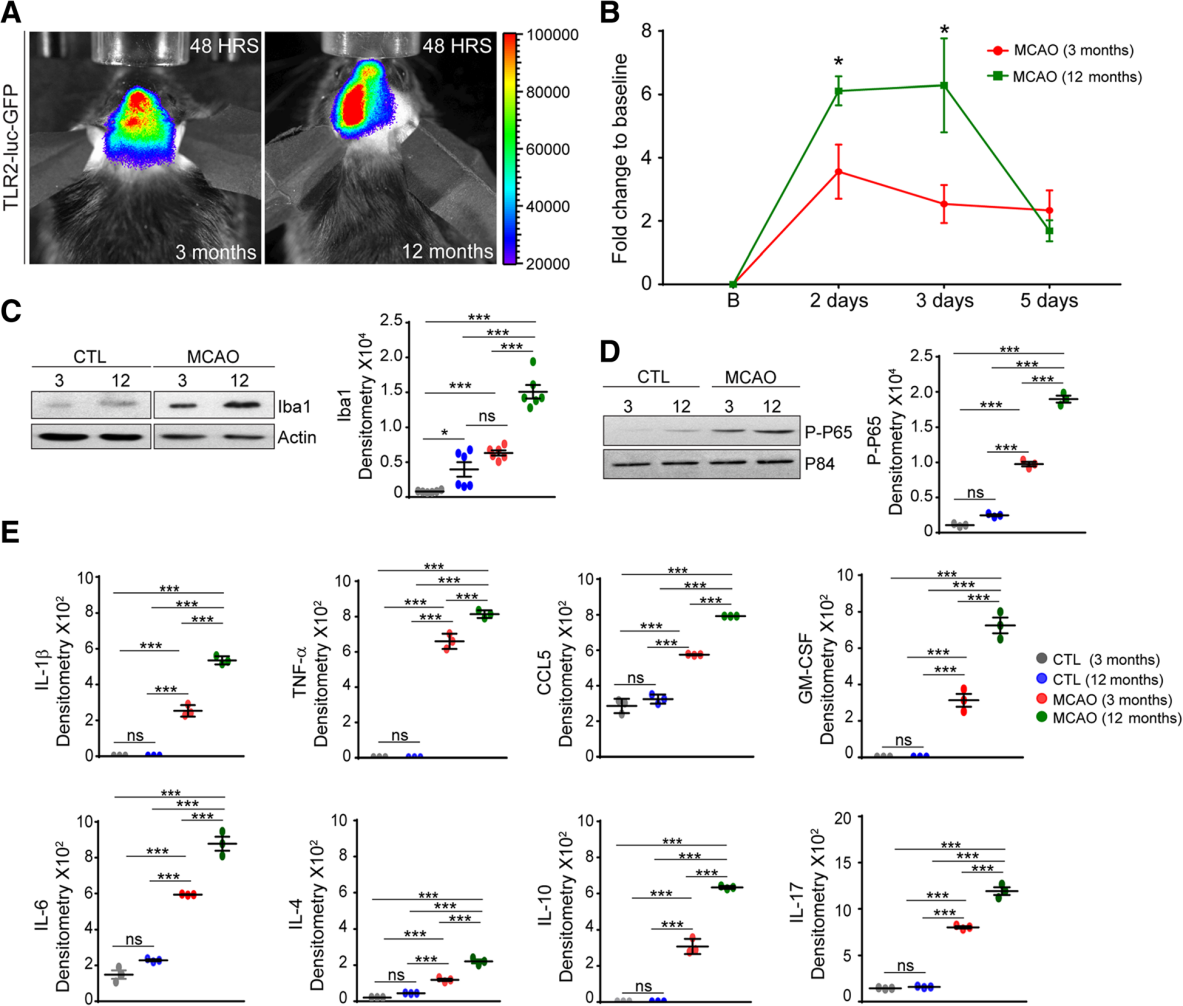
Cytoplasmic mislocalization of TDP-43 in neuronal and glial cells causes upregulation of inflammatory response 72 h after MCAO in aging. **a** Representative photographs 48 h after MCAO after real-time imaging of TLR2 induction. The color calibrations at the right are photon counts. **b** Data plotted was obtained measuring the photon emission. Red (3 month old) and green (12 month old) lines indicate TLR2 induction at different time points post MCAO. **c** Western blot of cortical lysates from 3- and 12-month-old mice using Iba1 in control and 72 h after MCAO reveals an increase in the levels of Iba1 in 12-month-old mice. Actin is used as loading control. **d** Western blot of nuclear lysates from 3- and 12-month-old wild-type mice using phospho-P65 in control and 72 h after MCAO reveals an increase in the levels of phospho-P65 in 12-month-old mice. P84 is used as loading control. **e** Levels of inflammatory cytokines like IL-1β, IL-4, IL-6, IL-10, IL-17, TNF-α, CCL5, and GM-CSF were significantly increased in 12-month-old mice 72 h after MCAO. Quantified data in the figure was presented as mean ± SEM and statistical significance between the groups was achieved using one-way ANOVA followed by Tukey’s multiple comparison test and depicted as ***p < 0.001, *p < 0.05

Microglial activation coupled with a marked induction of Toll-like receptor 2 (TLR2) are characteristic features of the brain response to ischemic injury [23]. Our previous reports have demonstrated that overexpression of TDP-43 in transgenic mice increases expression of inflammatory markers and TLR2 in glial cells [17, 24]; thus, we hypothesized that increase in TDP-43 levels and the formation of ubiquitin/TDP-43-positive inclusions observed predominately in 12-month-old mice would favor shift of microglial profiles toward pro-inflammatory phenotype resulting in an increase of the TLR2 response after stroke. To visualize microglial activation patterns and TLR2 response after MCAO, we took advantage of the TLR2-luc/GFP reporter mouse model previously generated and validated in our laboratory (Fig. 3a) [23]. In this in vivo model system, the reporter genes luciferase and GFP are co-expressed under transcriptional control of the murine TLR2 gene promoter thus allowing visualization of the luciferase signals from the brains of living mice [23]. Importantly, our previous studies have demonstrated that the TLR2 signal induction represents a valid readout measure of microglia activation after stroke [30–32]. As shown in Fig. 3b, we analyzed and compared the TLR2 signals normalized to a baseline value after stroke in young and old mice. The quantitative analysis of the TLR2 signals after MCAO revealed a significant increase in the TLR2 signal in 12-month-old mice when compared to 3-month-old mice. The differences were more pronounced in the acute phase of the response, i.e., 48–72 h after stroke (Fig. 3a, b). That microglial cells were more activated in the ischemic brains of 12-month-old mice was further confirmed by the quantitative analysis of the standard microglial marker Iba1. As shown in Fig. 3c, Western blot analysis of brain homogenates collected from 3- and 12-month-old mice in control conditions and 72 h after MCAO showed a marked increase in Iba 1 expression levels after stroke when compared to control conditions. The expression of Iba1 was strongly induced by MCAO in both age groups. However, a significantly higher induction of Iba1 was observed in the ischemic brains of the 12-month-old mice when compared to younger mice (Fig. 3c). Importantly when comparing the non-stroke controls, a baseline expression level of Iba1 was also significantly higher in 12-month-old controls animals when compare to younger animals. Together, these findings suggest the existence of age-dependent processes that are associated with an increase in inflammatory signaling in the brain.

We previously demonstrated that deregulation of TDP-43 observed in a mouse model of ALS/FTDL potentiates NF-κB-mediated pathogenic pathways [22]. We showed that TDP-43 may act as a co-activator of the P65 subunit of NF-κB and helps in the transcription of pro-inflammatory genes leading to release of inflammatory cytokines and causing inflammation-induced neurodegeneration [22]. The active form of P65, the phospho P65 (P-P65), can be used as an indicator of NF- κB-associated inflammation. We next examined whether NF-κB-mediated pathway is activated after stroke and whether the observed age-related increase in cytoplasmic TDP-43 is associated with an increase in P-P65. As shown in Fig. 3d, we quantified the P-P65 level in nuclear lysate from the brains of 3- and 12-month-old controls and acutely stroked mice (72 h post MCAO). The P-P65 levels were found to be significantly up-regulated in 12-month-old mice 72 h post MCAO when compared to 3-month-old mice in the same conditions (Fig. 3d). Although we observed a tendency, there was no significant difference in P-P65 level between 3- and 12-month-old control mice (Fig. 3d). To further asses the age-dependent immune profile of the brain after stroke, as previously described [33], we performed a multiplex cytokine array analysis. We measured expression levels of several pro- and anti-inflammatory cytokines in the brain. As shown in Fig. 3e, analysis of the inflammatory response in the brains of stroked mice revealed an increase in general immune response in the 12-month-old mice 72 h after MCAO. In fact, cytokine array data showed a significant increase in the levels of both the pro- and anti-inflammatory cytokines and chemokines like IL-1β, TNF-α, CCL5, GM-CSF, IL-6, IL-4, IL-10, and IL-17 in this specific group (Fig. 3e).

### Larger ischemic lesions and increased neuronal death are observed in 12-month-old mice

**Fig. 4:**
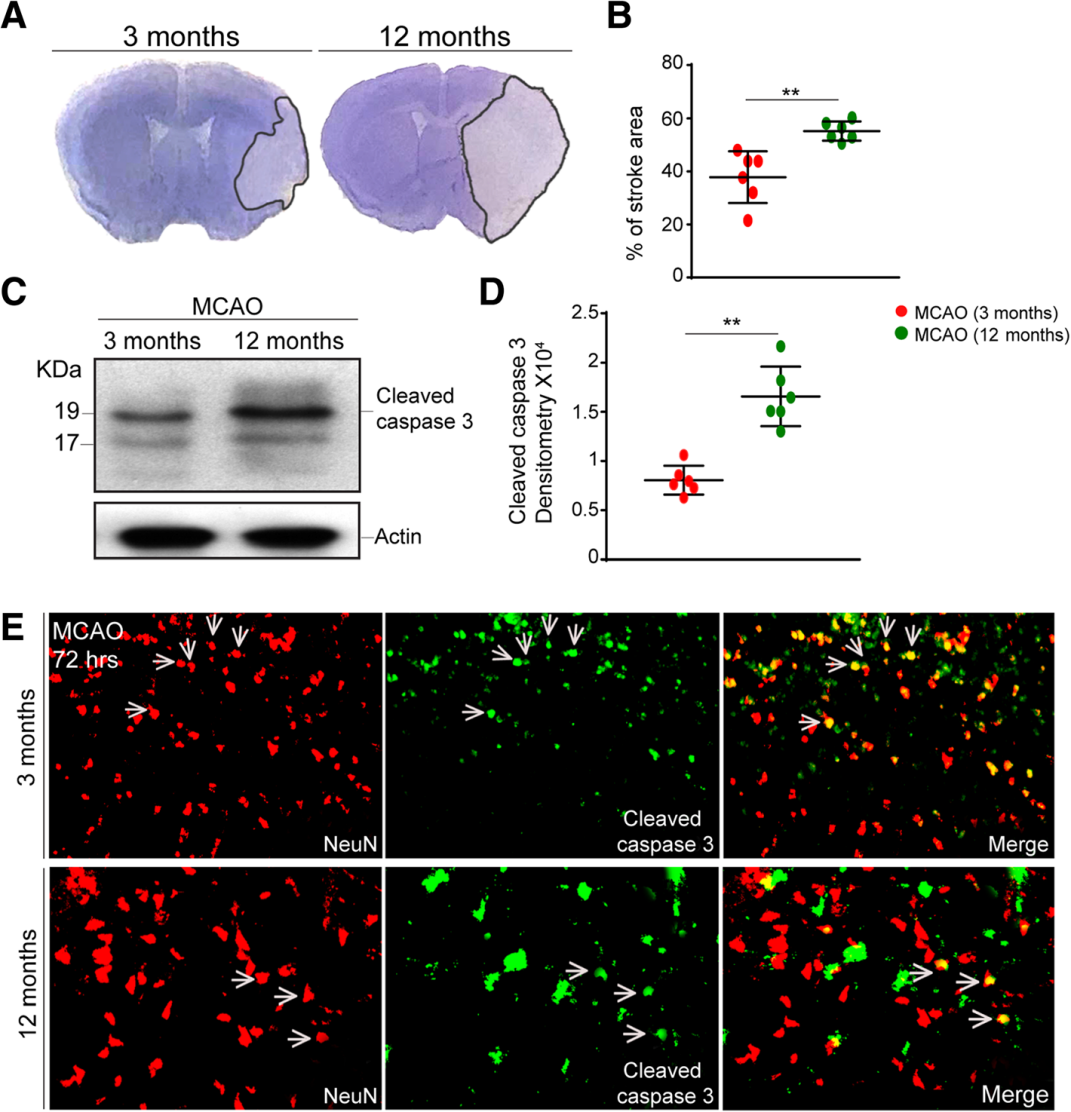
Increased stroke area and neuronal apoptosis was observed 72 h after MCAO in 12-month-old mice. **a** Representative images of cresyl violet staining showing stroke area in 3- and 12-month-old mice 72 h after MCAO. **b** Quantification of stroke area reveals an increase in the ischemic region in 12-month-old mice 72 h after MCAO. **c** Western blots of cytoplasmic lysates from 3- and 12-month-old mice using cleaved caspase-3 72 h after MCAO. **d** Normalized densitometry values of immunoblots 72 h after MCAO reveal a significant increase in the levels of cleaved caspase-3 in 12-month-old mice suggesting more tissue damage 72 h after MCAO. **e** Double immunofluorescence of the brain cortex sections of 3 and 12-month-old mice 72 h after MCAO using caspase-3 antibody (green) and NeuN (red) antibody show neuronal apoptosis in both age groups. Quantified data in the figure was presented as mean ± SEM and statistical significance between the groups was achieved using unpaired *t* test and depicted as ***p < 0.001, **p < 0.01

We next investigated to what extent the observed age-related deregulation of cytokine response after stroke affects evolution of the post-stroke ischemic injury. As previously described [33] and shown in Fig. 4a, we first measured and compared the size of the ischemic lesion in 3- and 12-month-old mice. The cresyl-violet-stained sections were analyzed 72 h after MCAO. In agreement with previous results, there was a significant, 17.4% increase in the size of ischemic lesion in the 12-month-old mice when compared to 3-month-old mice (Fig. 4a, b). As further shown in Fig. 4c, d, the observed increase in brain damage was accompanied by an increase in cellular death/apoptosis. Namely, a Western blot analysis of brain tissue homogenates from the ipsilateral/ischemic region of the brain showed a significant, twofold increase in the expression levels of the cleaved caspase-3 in the ischemic brains of 12-month-old mice, 72 h after MCAO (Fig. 4d). Next, a double immunofluorescence analysis revealed that the vast majority of the cleaved caspase-3 expressing cells were positive for neuronal marker NeuN (Fig. 4e). In accordance with our previous work [31, 34], this suggests that neurons are the principal cell type undergoing cellular stress and apoptosis after MCAO.

### TDP-43 cytoplasmic mislocalization exacerbates ischemic injury and neuroinflammation after stroke

**Fig. 5:**
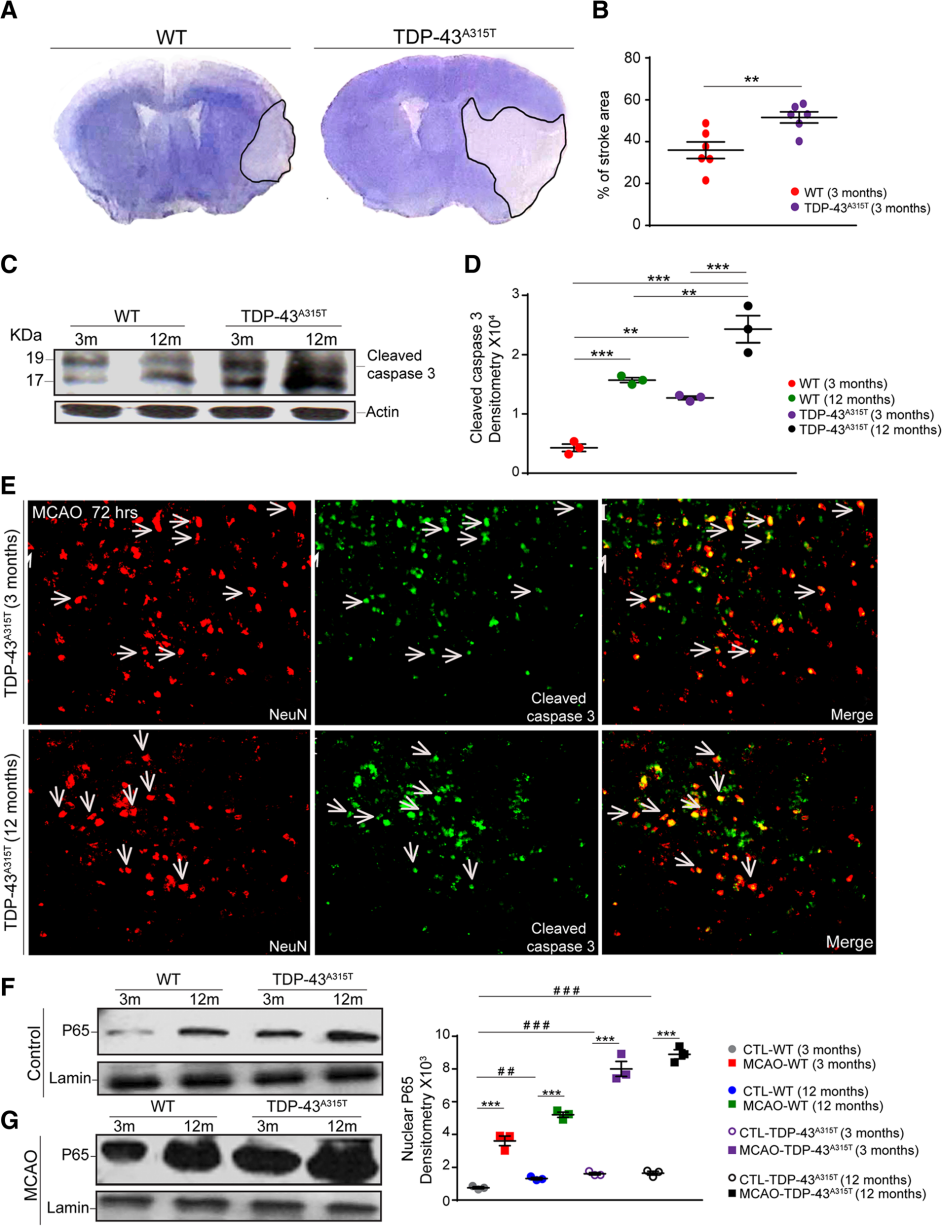
Overexpression of TDP-43 increases brain damage and inflammatory response after stroke. **a** Representative image of cresyl violet staining showing stroke area in 3-month-old WT and TDP-43^A315T^ 72 h after MCAO. **b** Quantification of stroke area reveals an increase in the ischemic lesion in TDP-43^A315T^ compared to WT mice. **c** Western blots of cytoplasmic lysates coming from 3- and 12-month-old WT and TDP-43^A315T^ mice using cleaved caspase-3. **d** Quantification of immunoblots 72 h after MCAO reveal a significant increase in the levels of cleaved caspase-3 in 12-month-old TDP-43^A315T^ mice when compared to the 3-month TDP-43^A315T^ or the 12-month-old WT control. **e** Double immunofluorescence of the brain cortex sections of 3- and 12-month-old TDP-43^A315T^ mice 72 h after MCAO using caspase-3 antibody (green) and NeuN (red) antibody show neuronal apoptosis in both age mice. **f** Expression of P65 by revealed by Western blot in control mice (3- and 12-month-old WT and TDP-43^A315T^). **g** Expression of P65 by revealed by Western blot 72 h after MCAO (3- and 12-month-old WT and TDP-43^A315T^). Normalized densitometry values of Western blot reveal a general significant increase in the levels of P65 after MCAO in either age group or mice. 3- and 12-month-old TDP-43^A315T^ show a higher P65 base level compared to the 3-month-old WT control. Quantified data in the figure was presented as mean ± SEM and statistical significance between the groups was achieved using unpaired *t* test and depicted as ***p < 0.001, **p < 0.01

Previous evidence suggests that cytoplasmic mislocalization of TDP-43 and formation of ubiquitinated aggregates is toxic to neurons and may enhance neuroinflammation [22, 24, 35]. Here, we hypothesized that age-related increase in the cytoplasmic TDP-43 observed in the brains of 12-month-old mice in control conditions and after stroke alters the immune microenvironment and may increase the susceptibility of neurons to ischemic damage. To test our hypothesis, we took advantage or the transgenic TDP-43 mice with moderate and ubiquitous overexpression of human TDP-43 (Tg line A315T). Importantly, at young age, starting at 2–3 months, these mice show an increase in cytoplasmic/ubiquitinated TDP-43 in neurons and glial cells, corresponding (resembling) to TDP-43 expression levels and/or expression patterns in aged, 12-month-old WT mice. In keeping with our hypothesis and as shown in Fig. 5a, b, the size of ischemic lesion was significantly increased in TDP-43 transgenic mice when compared to WT aged-matched littermates. Here, it is noteworthy that the observed increase in the size of ischemic lesion was corresponding to a size of ischemic lesion observed in 12-month-old mice (Figs. 3a and 4a). As shown by Western blot analysis, the cleaved caspase-3 expression levels were significantly increased in the ischemic brains of TDP-43 transgenic mice (Fig. 5c, d). A double immunofluorescence labeling for neuronal markers NeuN and cleaved caspase-3 revealed a marked co-localization of these two markers indicating an increase in the number of apoptotic neurons in the ischemic brains of TDP-43 transgenic mice (Fig. 5e). Next, we asked whether observed exacerbation of the ischemic injury and injury-induced neuronal apoptosis is caused by an increase in NF-κB-mediated inflammation. As described above (see Fig. 5f, g), we measured the levels of nuclear P65, an indicator of NF-κB-associated inflammation, in nuclear lysate from the brains of 3- and 12-month-old controls and TDP-43 transgenic mice in control conditions and following stroke (72 h post MCAO). As further shown in Fig. 5f, g, the expression of nuclear P65 is significantly increased in 12-month-old WT mice when compared to young WT controls. Interestingly, as shown in Fig. 5f, g, the expression of nuclear P65 is at comparable levels between old controls and 3-month-old TDP-43 transgenic mice thus suggesting a correlation of cytoplasmic TDP-43 levels and NF-κB activation.

### Increase in the cytoplasmic TDP-43 immunoreactivity in human stroke

**Fig. 6:**
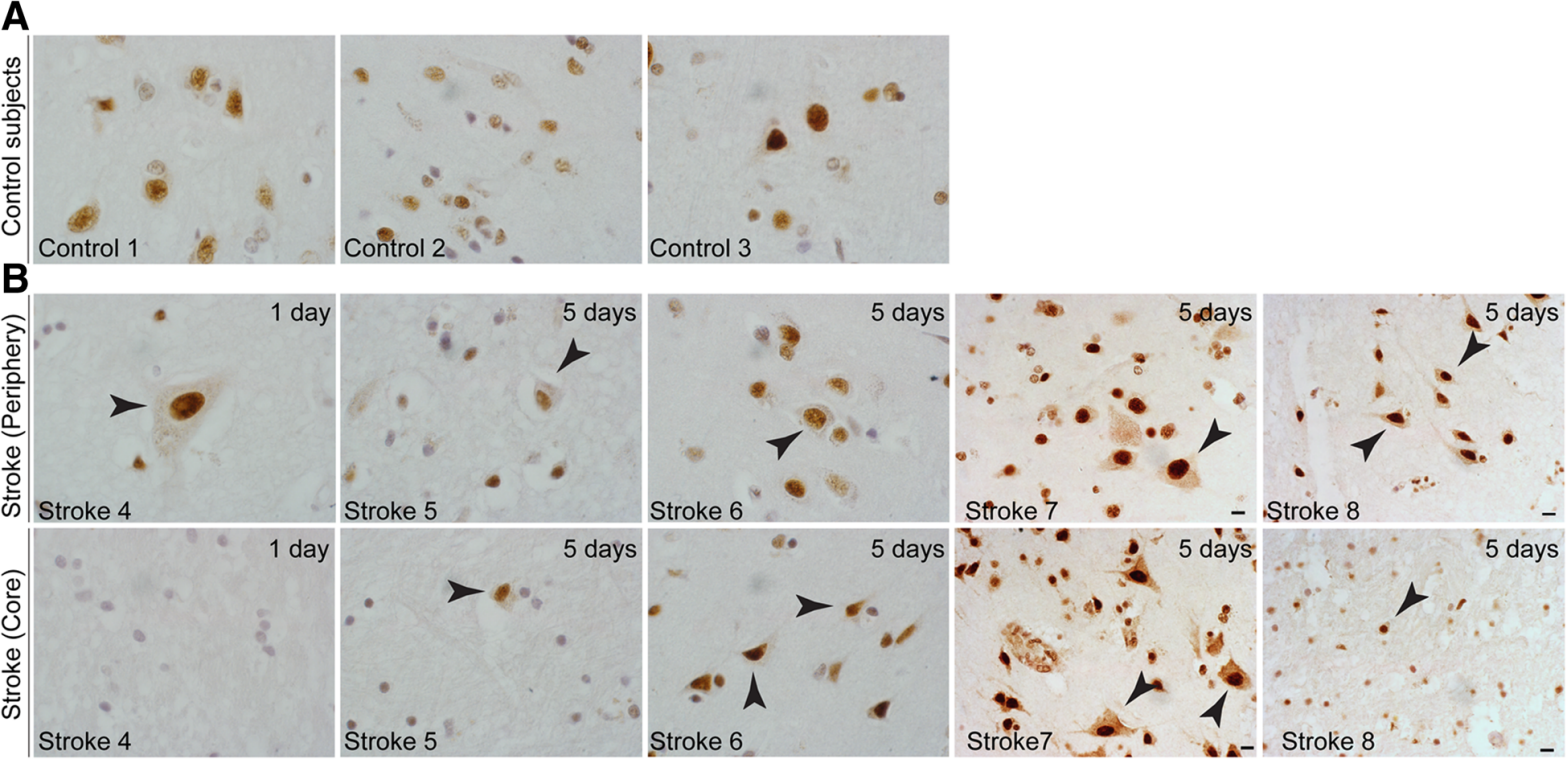
TDP-43 immunoreactivity in human stroke. **a** Immunohistochemistry of a control contralateral and non-affected hemisphere of the human subjects using anti human TDP-43 antibody reveals the localization of TDP-43 in the nucleus. **b** TDP-43 immunohistochemistry on cortical sections of human subjects died 1 day (stroke 4) and 5 days (stroke 5, 6, 7, 8) after ischemic stroke reveals the localization of TDP-43 in the cytoplasm of neurons in the periphery and core regions of the ischemia, which is indicated with arrow marks in black. Scale bar represents 20 μm

At present, it remains unclear to what extent and/or whether TDP-43 pathology is associated with ischemic injury in human stroke. To address this issue, we performed immunohistochemistry analyses of the post-mortem post-stroke brain tissue autopsied at 1 to 5 days after stroke. The analysis was performed by focusing on two distinct region of the ischemic lesion, the peri-infarct and core region (cortical sections) and compared with corresponding controls. The analysis was performed using a human anti-TDP-43 antibody (Fig. 6a, b). As shown in Fig. 6a, control sections stained with an anti-human TDP-43 antibody displayed well-circumscribed and positively stained nuclei, while the cytoplasmic compartment was almost completely devoid of TDP-43 immunoreactivity. Interestingly, resembling the TDP-43 expression patterns following single traumatic brain injury in humans [20], the TDP-43 staining following acute ischemic stroke revealed increased immunoreactivity within the cytoplasm and in some cases extending into processes (Fig. 6b). The increase in the cytoplasmic TDP-43 immunoreactivity was more prominent at day 5 after stroke. Hence, ischemic stroke in humans is associated with an increase in TDP-43 immunoreactivity in the cytoplasmic compartment. However, unlike the TDP-43 expression patterns observed in the chronic TDP-43 proteinopathies, in stroke, the nuclei remain positively stained for TDP-43.

## Discussion

The work presented here provides an important in vivo evidence for a pathogenic role of TDP-43 in stroke. Based on the results presented in this study, we propose here that age-related deregulation of TDP-43 exacerbates inflammation and ischemic injury and may contribute to post-stroke neurodegenerative processes. By investigating TDP-43 expression patterns after stroke in young and 12-month-old mice, we showed (i) a marked increase and accumulation of TDP-43 in cytoplasmic compartment in neurons and microglia (ii) levels of mislocalized/cytoplasmic TDP-43 and its cleaved pathogenic fragments TDP-35 and TDP-25 were more elevated in aged mice, (iii) observed deregulation of TDP-43 was associated with an increase in post-stroke inflammation and larger infarctions, (iv) overexpression of TDP-43 further exacerbated ischemic injury and markedly enhances inflammation via activation of NF-κB, and (v) mislocalization of TDP-43 into cytoplasmic compartment occurred also in human stroke.

Cerebral ischemia is characterized by a marked acute and chronic inflammatory response. We and others have shown that post-stroke inflammation may have a marked chronic component that may last several months following an initial ischemic event and may contribute to development of the chronic brain injury [23, 36]. However, the molecular mechanisms driving the long-lasting post-stroke inflammation, and potentially leading to a neurodegeneration, remain elusive. Based on the results described in the current study, we hypothesized that the age-related accumulation of TDP-43 in the cytoplasm (see Fig. 2) may drive chronic inflammation after stroke and thus contribute to ischemic injury.

To date, little is known about the role of TDP-43 in the pathogenesis of stroke. The alterations in TDP-43 expression in response to ischemic injury have been recently described by Kanazawa and colleagues in an acute rat model of ischemic injury [37]. However, the study has been limited to a 24 h after stroke time period [37]. Our findings are generally in agreement with the initial report. However, we observed a long-lasting (up to 30 days post MCAO) cytoplasmic accumulation/deregulation of TDP-43 after stroke (Fig. 1). In addition, our study extends the initial report in several ways. First, in keeping with recent report of the TDP-43 contribution in aging, we investigated the expression patterns of the TDP-43 after stroke in two different age groups (3 and 12 month old). Importantly, our results revealed markedly increased accumulation of the cytoplasmic TDP-43 in older mice. The fact that in older mice the cytoplasmic TSP-43 was present even at the baseline levels may have significantly affected the initial brain response to ischemic injury. Indeed, the levels of pathogenic TDP-35 and TDP-25 fragments after stroke were significantly increased in 12-month-old mice when compared to young animals, thus further suggesting a strong component of aging in TDP-43 mediated pathology. As previously mentioned, TDP-43 expression is normally restricted to nucleus and has a role in the regulation of gene transcription, mRNA splicing, mRNA stability, and transport. However, in pathological conditions, TDP-43 becomes mislocalized to the cytoplasm of both neurons and glial cells and cleaved by the caspases into 35 kDa and 25 kDa fragments to form potentially pathogenic aggregates [38].

Here, we presume that cytoplasmic accumulation of TDP-43 during ischemic stroke occurred because of deregulation of its nuclear import. In fact, the C-terminal fragments of TDP-43 that are formed during aging/stress lack functional nuclear localization signal [39]. Cytoplasmic accumulation of TDP-43 protein was reported in different neurodegenerative diseases [37, 40] [17]. For example, development of many age-related pathological and biochemical changes like formation of C-terminal TDP-43 fragments, TDP-43/ubiquitin aggregates, and neuroinflammation have been reported in mouse model of ALS [41]. Indeed, in TDP-43 proteinopathies, dying neurons display the presence of ubiquitin inclusions as the ubiquitin-dependent protein degradation pathways were hampered [42]. Another important feature of TDP-43 proteinopathies is a presence of phosphoTDP-43 aggregates. Evidence suggests that casein kinase 1 phosphorylates TDP-43 to form insoluble phosphoTDP-43 aggregates in most of the TDP-43 proteinopathies [43]. To investigate the formation and presence of insoluble phosphoTDP-43 aggregates after ischemic injury and in aging, we collected urea-SDS insoluble fraction and performed immunoblots. Surprisingly, we did not detect phosphorylated TDP-43 aggregates in any of tested age groups after stroke, suggesting that TDP-43 pathology has distinct molecular signature after stroke when compared to chronic neurodegenerative TDP-43 proteinopathies.

Another hallmark of the brain response to ischemic injury and neurodegeneration is activation of glial cells. Indeed, previous studies reported TDP-43 cytoplasmic inclusions glial cells in the spinal cords of the ALS patients [15] while in vitro studies using microglia and astrocyte culture exhibits TDP-43 mislocalization in induced neuroinflammatory conditions [41]. Furthermore, our previous studies have demonstrated that binding of its N-terminal and RRM1 domains to p65, TDP-43 acts as co-activator of p65 NF-kB thus leading to enhanced activation of the NF-κB pathways [22]. Importantly, NF-κB may interact with specific proteins and DNA sequences to trigger inflammation and ischemic injury-induced neuronal apoptosis [44]. It was reported that NF-κB translocate to the nucleus from the cytoplasm in cerebral ischemic injury [45]. Indeed, in the present study, we demonstrated a TDP-43-mediated deregulation and activation of NF-κB pathway. We measured the nuclear phosphorylation levels of P65 subunit of NF-κB after stroke in both 3- and 12-month-old mice. We observed that after the ischemic injury, the phospho-P65 subunit travels to the nucleus from the cytoplasm in both groups. There was an increased amount of phospho-P65 subunit in the nucleus of 12-month-old mice compared to 3-month-old mice 72 h after MCAO. In an additional *proof*-*of*-*concept* experiments using TDP-43 A315T transgenic mice that overexpress TDP-43 in the cytoplasm, at the similar levels as 12-month-old WT mice, we observed comparable levels of nuclear phosphorylation levels of P65 subunit of NF-κB after stroke.

An important question here is how deregulation of TDP-43 affects neuronal survival and microglia-neuron crosstalk after ischemic injury. Substantial loss of nuclear TDP-43 in ischemic neurons may lead to nuclear dysfunction. Indeed, a series of in vitro experiments showed that TDP-43 depletion/silencing may cause disturbance in cell cycle leading to cell death [46]. Additional evidence suggests that TDP-25 fragments formed in the cytoplasm of neurons can gain toxic functions and thus cause tissue damage albeit the mechanism remains unknown [47]. Importantly, both of these neuropathological features were detected in ischemic brain tissue and affected neurons. Therefore, we examined whether the observed age-related TDP-43 deregulation may lead ultimately lead to more neuronal damage. Indeed, we found a significantly larger infarctions in the 12-month-old than 3-month-old mice 72 h after MCAO as well as an increased in cleaved caspase-3 levels in aging brain after 72 h post ischemia, while the analyses of the ischemic lesion in the context of TDP-43 cytoplasmic overexpression also showed direct correlation between TDP-43 expression levels and the size of the ischemic lesion. The important question that has been raised here is whether the observed deregulation in TDP-43 expression patterns is present in human stroke? Importantly, analyses of the post-mortem and post-stroke brain tissues revealed the presence of the cytoplasmic TDP-43 immunoreactive structures in human stroke resembling those observed after single brain trauma [20]. Together, our results suggest that deregulation of TDP-43 may represent a converging pathogenic pathway that drives neuroinflammation following acute brain injuries and in chronic neurodegeneration.

## Conclusion

In conclusion, ischemic injury is associated with a marked and age-related deregulation of TDP-43. Further, we observed a significant increase in TDP-43-mediated modulation of NF-κB neuroinflammation leading to increase in neuronal injury and neurodegeneration after stroke. Although the stroked tissue did not display the full neuropathological feature associated with chronic TDP-43 proteinopathies, i.e., the presence of highly phosphorylated TDP-43 aggregates and complete depletion of nuclear TDP-43, we showed that ischemic injury may cause a long-lasting cytoplasmic accumulation of TDP-43. Thus, it is possible that deregulation of TDP-43 observed after the initial ischemic event may drive chronic post-stroke inflammatory response and may represent an age-related risk factor for development of neurodegenerative disorders. Based on our results, we propose that therapies targeting mislocalized/cytoplasmic TDP-43 may have a potential to attenuate post-stroke inflammation and ischemia-induced neuronal injury.

## Additional file

Additional file 1: **Table S1**. Details and pathological features of the stroke subjects presented in Fig. 6. (PDF 100 kb)

## Abbreviations

CCD: Charge coupled device
CCL5: Chemokine C-C motif ligand 5
GM-CSF: Granulocyte-macrophage colony stimulating factor
MCAO: Middle cerebral artery occlusion
NF-κB: Nuclear factor kappa B
TDP-43: Transactive response (TAR) DNA binding protein 43 (TDP-43)
TLR2: Toll-like receptor 2
WT: Wild type
