# Supplementary material for: Age-related deregulation of TDP-43 after stroke enhances NF-κB-mediated inflammation and neuronal damage
Source: J Neuroinflammation. 2018 Nov 9;15:312. doi: 10.1186/s12974-018-1350-y (PMC6230239; doi:10.1186/s12974-018-1350-y)
Supplement: Supplementary file 1 — Table S1. Details and pathological features of the stroke subjects presented in Fig. 6. (PDF 100 kb) [file 12974_2018_1350_MOESM1_ESM.pdf]

Supplemental Table 1

Cod_BTN	Case	Age	Gender	Admission NIHSS	Vascular territory	Intracranial oclusion site	Acute revascularization therapy	Hemorrhagic transformation	Etiology	Exitus (days after onset)	Exitus to necropsy timelapse	Acute infection
BK1823	IC6	63	Woman	20	Right MCA infarct	Right M1	Yes	No	Undetermined	1 day	2 h	Yes
BK1560	IC1	88	Woman	19	Right MCA infarction Left MCA and IH2 recurrence	Right and Left M1	Yes	No	Cardio-embolic	5 days	5:45 h	No
BK1563	IC2	79	Man	4	Left MCA infarct	Left M2	None	Parieto-occipital parenchymal hematoma type 2	Large vessel disease	5 days	4:35 h	No

Details and pathological features of the stroke subjects presented in figure 6
